# Outcomes of Adrenalectomy and the Aldosteronoma Resolution Score in the Black and Hispanic Population

**DOI:** 10.1007/s00268-021-05967-y

**Published:** 2021-02-07

**Authors:** Gustavo Romero‐Velez, Amanda M. Laird, Manuel E. Barajas, Mauricio Sierra-Salazar, Miguel F. Herrera, Steven K. Libutti, Michael K. Parides, Xavier Pereira, John C. McAuliffe

**Affiliations:** 1 General Surgery, Department of Surgery, Montefiore Medical Center, The Bronx, NY USA; 2 Section of Endocrine Surgery, Rutgers Cancer Institute of New Jersey, New Brunswick, NJ USA; 3 Division of Endocrine Surgery, Department of General Surgery, Instituto Nacional de Ciencias Medicas Y Nutricion Salvador Zubiran, Mexico City, Mexico; 4 Rutgers Cancer Institute of New Jersey, New Brunswick, NJ USA; 5 Department of Cardiothoracic and Vascular Surgery, Montefiore Medical Center, The Bronx, NY USA; 6 Surgical Oncology, Department of Surgery, Montefiore Medical Center, 1865 Eastchester Rd. Suite 2S7, Bronx, NY 10461 USA

## Abstract

**Background:**

Outcomes after adrenalectomy in patients with primary aldosteronism (PA) are variable. The aldosteronoma resolution score (ARS) uses preoperative variables to calculate a score that identifies those patients that are more likely to have resolution of hypertension after adrenalectomy. We aim to determine the efficacy of adrenalectomy and whether the ARS accurately predicts clinical success in a Black and Hispanic population.

**Methods:**

We reviewed patients who underwent adrenalectomy for PA from 2004 to 2018 at two academic centers treating primarily Hispanic and Black patients. Postoperative outcomes were evaluated based on the primary aldosteronism surgical outcome consensus criterion. Retrospectively, the accuracy of ARS was determined by a receiver operating characteristic curve and the area under the curve (AUC).

**Results:**

Forty-three Hispanic and 10 Black patients underwent adrenalectomy for PA. Twenty-two patients (41.5%) had complete clinical success. Variables associated with complete clinical success in the univariate analysis were female gender (*p* = 0.026), younger age (*p* = 0.001), lower preoperative aldosterone (*p* = 0.035), lower preoperative systolic blood pressure (*p* = 0.001), fewer number of preoperative antihypertensive medications (*p* = 0.007) and a higher ARS (*p* = 0.003). On multivariate analysis, only fewer number of preoperative antihypertensive medications was independently associated with complete clinical success (*p* = 0.026). The AUC of the ARS was 0.746.

**Conclusion:**

The rate of clinical success from adrenalectomy is good for Hispanic and Black patients with PA. Our analysis shows that the ARS is an accurate test of clinical success in Hispanic and Black patients. The ARS may be utilized preoperatively to frame expectations after adrenalectomy in these populations.

## Introduction

Primary aldosteronism (PA) is present in up to 33% of patients with secondary hypertension (HTN) [1]. It is characterized by renin-independent excess aldosterone which leads to drug-resistant hypertension and hypokalemia [2]. The source of aldosterone excess in PA may be from one adrenal gland as a result of an aldosterone-producing adenoma (APA) or unilateral hyperplasia. Alternatively, PA can be caused by bilateral adrenal hyperplasia, also known as idiopathic hyper-aldosteronism (IHA). This distinction is important as IHA is typically treated with mineralocorticoid receptor antagonists while unilateral hypersecretion may be managed with adrenalectomy [3]. Resolution of HTN after adrenalectomy is variable with some groups responding better than others.

Zarnegar et al. [4] developed the aldosteronoma resolution score (ARS) to preoperatively predict complete resolution of secondary HTN due to PA in surgically treated patients [4]. This scoring system uses 4 preoperative clinical variables (body mass index (BMI), gender, number of antihypertensives, and duration of HTN), giving points for each variable. Those with ARS scores of 4–5 have a higher chance complete clinical success (80%) compared to those with ARS scores of 1–2 (13.7%). This scoring system has been validated in different populations and was found to have good accuracy [5]–[9]. However, Hispanic and Black populations were not well represented in these cohorts. In Hispanic and Black populations, PA has been observed in 7.2% of hypertensive patients [10]–[12].

However, treatment of secondary HTN in Hispanic and Black patients presents a particular challenge. These cohorts have a higher prevalence of essential HTN as well as non-adrenal-related secondary HTN compared to other races. This variation is often attributed to genetic and socioeconomic differences in these groups [11, 13]. Therefore, there may be a clinical success disparity in these populations treated with adrenalectomy for PA. Clinical success following adrenalectomy for PA in the Black and Hispanic population remains unclear. In addition, factors that may predict clinical success in this population are not well-defined.

We sought to determine the clinical success of adrenalectomy for Hispanic and Black patients with PA. We then evaluated the ARS as a model for predicting clinical success in this population.

## Material and methods

### Patient cohort

We reviewed medical records of consecutive patients who underwent adrenalectomy for PA from 2004 to 2018 at Montefiore Medical Center (MMC) (Bronx, NY, USA) and Instituto Nacional de Ciencias Medicas y Nutricion Salvador Zubiran (INNSZ) (Mexico City, Mexico). Patients of other races were excluded. The Institutional Review Board of both institutions approved this study.

### Data collection

Patient baseline characteristics included age, gender, race, duration of HTN, BMI, number of antihypertensive medications, initial blood pressure, size of the tumor on imaging, and use of adrenal vein sampling (AVS). Biochemical data included serum potassium, aldosterone levels and plasma renin activity. Postoperative data collected include serum potassium, aldosterone, and renin levels when available, systolic and diastolic blood pressure recordings, and number of antihypertensive medications.

### Predictive model

The ARS for each patient in our cohort was retrospectively calculated using preoperative data as originally described by Zarnegar et al. [4]. One point was given for each of the following variables: BMI ≤ 25 kg/m^2^, duration of HTN ≤ 6 years and female gender. Two points were given if patients were taking ≤ 2 antihypertensive medications.

### Definitions

Hypertension was defined as a systolic blood pressure (SBP) ≥ 140 mmHg or diastolic blood pressure (DBP) ≥ 90 mmHg. PA was determined by the presence of persistent hypertension with or without hypokalemia (< 3.6 mEq/L), with increased serum aldosterone concentration (> 15 ng/dL), suppressed plasma renin activity (< 1 ng/mL/hr) and aldosterone-to-renin ratio (ARR) (> 20). Unilateral adrenal disease was confirmed by cross-sectional imaging in patients not undergoing adrenal vein sampling (AVS). A proportion of a patients underwent AVS to subtype disease and confirm laterality.

Postoperative clinical success was defined according to the primary aldosteronism surgical outcome (PASO) consensus criterion [14]. Complete clinical success was defined as normal blood pressure (SBP < 140 mmHg, DBP < 90 mmHg) without the aid of antihypertensive medication. Partial clinical success was defined as the same blood pressure before surgery but with less antihypertensive medication or a reduction in blood pressure with either the same amount or less antihypertensive medication. Absent clinical success was defined as unchanged or increased blood pressure with either the same amount or an increase in antihypertensive medication postoperatively. Based on these definitions, groups were created using the information collected on the last available follow-up. Complete biochemical success was defined by PASO as plasma aldosterone < 5 ng/dL, however, this was not assessed during our study given incomplete postoperative aldosterone measurements.

### Statistics

Statistical analysis was performed using SPSS Statistics 22.0 (IBM Corp., Armonk, NY, USA). The Student's T‐test and chi-squared test were used for continuous and categorical variables, respectively. The Fischer exact test was used for categorical variables when an observed value was < 5. Statistical significance was considered at* P* < 0.05. Those variables with statistical significance were included in a logistic regression model to generate a multivariate analysis. Results are reported as median and range unless otherwise stated. We modeled the receiver operating characteristic (ROC) curve and the area under the curve (AUC) to illustrate the accuracy of the ARS in predicting clinical success of adrenalectomy for PA. Good predicting accuracy was considered with an AUC greater than 0.70 [15].

## Results

Fifty-seven consecutive patients with PA underwent adrenalectomy between 2004 and 2018 between the two institutions (29 patients (50.9%) from MMC and 28 patients (49.1%) from INNSZ). Fifty-three patients were included in the final analysis; 3 patients were excluded as their race was not Black nor Hispanic and one was excluded as the final pathology showed adrenocortical carcinoma. The patients’ clinical characteristics are displayed in Table 1.

**Table 1:** Characteristics of included patients

	Total cohort (*n* = 53)	Hispanic (*n* = 43)	Black (*n* = 10)
	100%	81.1%	18.9%
	Mean ± SD (range)	Mean ± SD (range)	Mean ± SD (range)
Age, years	44 ± 13 (18–73)	43 ± 12 (18–66)	49 ± 13 (36–73)
BMI	28 ± 5 (20–46)	27 ± 5 (20–42)	30 ± 7 (20–46)
Duration of HTN, years	4.2 ± 4.6 (1–17)	3.8 ± 4.2 (1–17)	6.8 ± 6.4 (1–15)
Tumor size, mm	9 ± 10 (5–47)	7 ± 9 (6–47)	16 ± 8 (5–33)
ARS	2.5 ± 1.6 (0–5)	2.5 ± 1.5 (0–5)	2.0 ± 1.9 (0–5)
Follow-up, months	9 ± 12 (1–84)	9 ± 13 (1–84)	10 ± 8 (1–24)
Female	54.7% (29/53)	53.5% (23/43)	54.5% (6/10)
Male	45.3% (25/53)	46.5% (20/43)	45.5% (5/10)
Pre-Op AVS	41.5% (22/53)	27.9% (12/43)	100% (10/10)

*BMI*: Body mass index, *HTN*: Hypertension, *ARS*: aldosteronism resolution score, *AVS*: adrenal vein sampling

The majority of the cohort was Hispanic (81.1%). There were 29 females (54.7%). The median age and BMI were 43 years (18–73) and 27 kg/m^2^ (20–46), respectively. The median duration of HTN was 2.3 years (1–17). Median preoperative systolic and diastolic BP was 150 mmHg (110–249) and 98 mmHg (70–151) on 3 (1–8) antihypertensives.

Median preoperative aldosterone, renin, ARR and potassium were 39 ng/dL (20–759), 0.2 ng/mL/hr (0.04–2.8), 256 (20–6800), and 3.4 mEq/L (2.49–5.0), respectively. On cross-sectional imaging, the left adrenal was most commonly affected (60.4%) and the mean tumor size was 9 mm (5–47). Forty-two percent of patients underwent AVS. All these patients were cared for at MMC. Fifty-one out of 53 (96.2%) of the adrenalectomies were completed laparoscopically. On pathologic examination, 46 (86.8%) were adenomas, and 7 (13.2%) were hyperplasia.

Postoperative median potassium, aldosterone, SBP, and DBP were 4.53 mEq/L (3.2–5.8), 5 ng/dL (1–26), 120 mmHg (90–153), and 80 mmHg (50–100), respectively, while on a median of 1 (0–5) antihypertensive. Importantly, while there was complete postoperative SBP, DBP and medication data for all patients, postoperative potassium and aldosterone were available for only 49 and 27 patients, respectively. Adrenalectomy for PA improved the SBP (*p* < 0.001), DBP (*p* < 0.001), number of antihypertensives taken (*p* < 0.001), serum potassium (*p* < 0.001) and serum aldosterone (*p* = 0.003) as compared to their counterpart preoperative recordings (Table 2).

**Table 2:** Preoperative vs. postoperative outcomes of entire cohort

	Pre-op	Post-op*	*p*-value
	Mean ± SD (range)	Mean ± SD (range)	
Average number of antihypertensives	2.9 ± 1.5 (1–8)	1 ± 1.2 (0–5)	< 0.001
Mean systolic BP, mmHg	155 ± 27 (110–249)	123 ± 14 (90–162)	< 0.001
Mean diastolic BP, mmHg	98 ± 16 (70–151)	78 ± 10 (50–100)	< 0.001
Mean potassium, mEq/L	3.5 ± 0.6 (2.49–5.0)	4.5 ± 0.5 (3.2–5.8)	< 0.001
Mean aldosterone, ng/dL	89 ± 148 (20–759)	6.4 ± 6.9 (1–26)	0.003
Mean renin, ng/mL/hr	0.3 ± 0.4 (0.04–2.8)	NA	NA
Mean ARR	525 ± 1018 (20–6800)	NA	NA

*BP*: Blood pressure; *ARR*: Aldosterone-to-renin ratio; *NA*: Not available

^*^data missing from 4 and 26 patients for potassium and aldosterone, respectively

### Clinical success

**Fig. 1:**
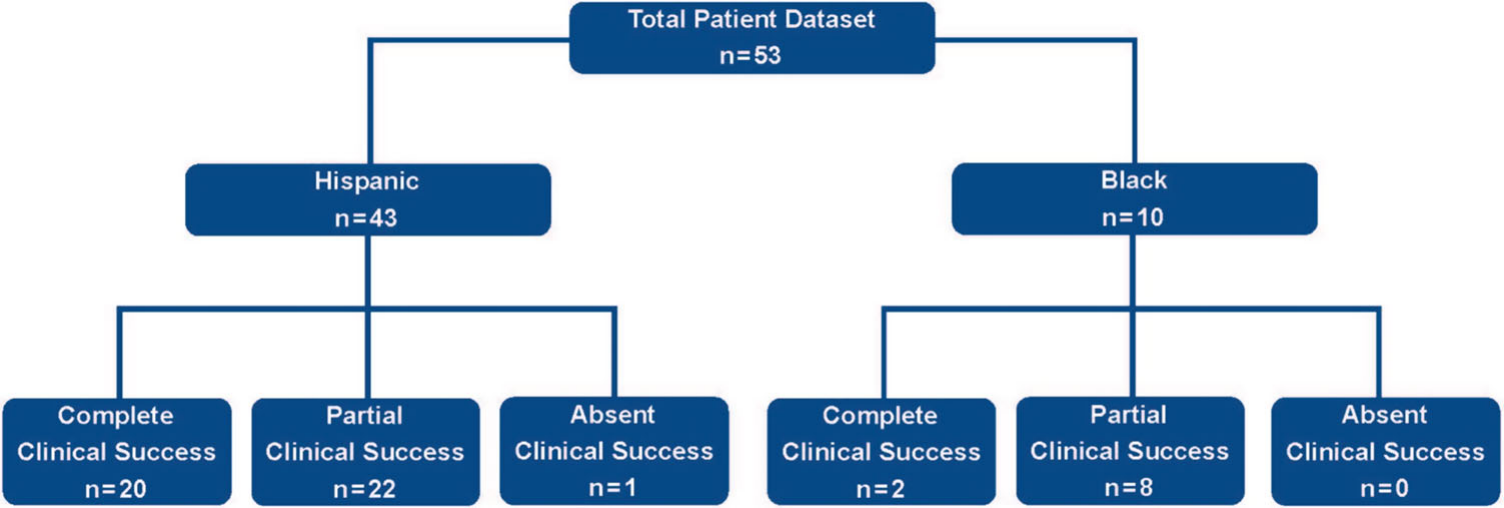
Composition of the cohort broken down by race followed by clinical response

Median follow-up was 6 months (1–84), at which time 48 patients (90%) had improvement in their serum potassium (4.53 mEq/L (3.2–5.8)). Twenty-two patients (41.5%) had complete clinical success and 30 (56.6%) had partial clinical success. Therefore, a combined benefit of 98.1% following adrenalectomy for PA was seen in our cohort. Only, one patient had absent clinical success (1.9%). Twenty percent of Black patients achieved complete clinical success (2/10), while 46% of Hispanic patients (20/43) achieved complete clinical success (Fig. 1). This ethnic difference in complete clinical success was not statistically significant (*p* = 0.166).

### Predictors of clinical success and ARS

**Fig. 2:**
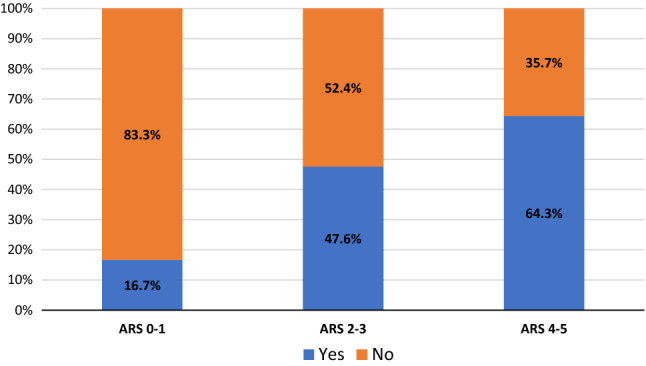
Percentage of patients with complete clinical success based on ARS

The proportion of patients with complete clinical success was 9/14 (64.3%) with an ARS 4–5, 10/21 (47.6%) with an ARS 2–3 and 3/18 (16.7%) for those with an ARS 0–1 (Fig. 2).

In univariate analysis, female gender (*p* = 0.026), younger age (*p* = 0.001), lower preoperative aldosterone (*p* = 0.035), lower preoperative systolic blood pressure (*p* = 0.001), fewer number of preoperative antihypertensives (*p* = 0.007) and higher ARS (*p* = 0.003) were associated with complete clinical success (Table 3). On multivariate analysis, fewer number of preoperative antihypertensive medications was independently associated with complete clinical success (*p* = 0.026) (Table 4).

**Table 3:** Univariate analysis of patient characteristics between patients with complete clinical success and incomplete clinical success (partial and absent grouped together)

	Complete clinical success (*n* = 22)	Partial/absent clinical success (*n* = 31)	*p-*value
*Clinical characteristics*			
Mean age, years	37.6 ± 12.2	49.0 ± 11.5	0.001
Mean BMI	26.9 ± 4.8	28.8 ± 5.9	0.210
Mean duration of HTN, years	3.1 ± 3.0	5.2 ± 5.6	0.136
Mean ARS	3.2 ± 1.3	1.9 ± 1.6	0.003
% Female	72.7% (16/22)	41.9% (13/31)	0.026
% Male	27.2% (6/22)	58.1% (18/31)	
% Hispanic	46.5% (20/43)	53.5% (23/43)	0.166
% Black	20% (2/10)	80% (8/10)	
% Patients Undergoing AVS	31.8% (7/22)	48.4% (15/31)	0.228
*Preoperative data*			
Antihypertensives, average number	2.2 ± 1.1	3.4 ± 1.6	0.007
Mean tumor size, mm	8.8 ± 12.3	9.7 ± 8.6	0.742
Mean SBP, mmHg	140.8 ± 19.4	164.4 ± 28.4	0.001
Mean DBP, mmHg	93.4 ± 15.8	101.3 ± 16.1	0.082
Mean potassium, mEq/L	3.47 ± 0.49	3.57 ± 0.61	0.558
Mean aldosterone, ng/dL	44.3 ± 37.9	123.5 ± 188.9	0.035
Mean renin, ng/mL/hr	0.20 ± 0.15	0.39 ± 0.54	0.127
*Final Histology*			
% Adenoma	95.5% (21/22)	80.6% (25/31)	0.218
%Hyperplasia	4.5% (1/22)	19.4% (6/31)	
*Postoperative outcomes*			
Antihypertensives, average number	0	1.7 ± 1.1	< 0.001
Mean SBP, mmHg	115.3 ± 8.6	128.4 ± 15.5	< 0.001
Mean DBP, mmHg	73.7 ± 6.7	80.9 ± 11.9	0.013
Mean potassium, mEq/L	4.64 ± 0.42	4.52 ± 0.49	0.372
Mean aldosterone, ng/dL	5.7 ± 6.4	7.1 ± 7.6	0.599

*BMI*: Body mass index; *HTN*: Hypertension, *SBP*: Systolic blood pressure; *DBP*: Diastolic blood pressure; *ARS*: Aldosteronism resolution score; *AVS*: Adrenal vein sampling

**Table 4:** Multivariate analysis of patient characteristics and complete clinical success

Variable	Odds ratio (95% CI)
Gender	0.4 (0.09–2.6)
Age	1.05 (0.99–1.13)
Preop aldosterone	1.00 (0.99–1.02)
No. antihypertensive medications preoperative	2.30 (1.1–4.77)
SBP preoperative	1.05 (0.99–1.09)

*CI*: Confidence interval; *SBP*: Systolic blood pressure

**Fig. 3:**
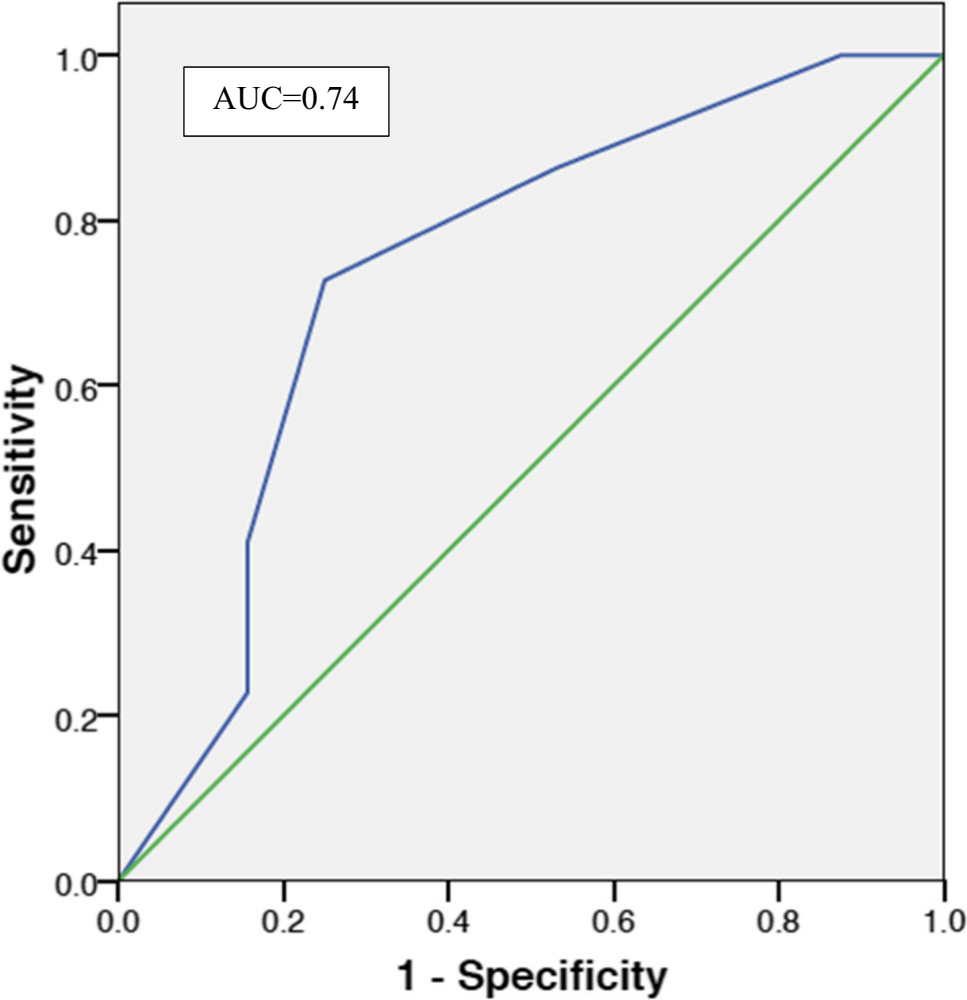
ROC Curve of the ARS applied to our patient cohort

Based on preoperative clinical characteristics, the ARS was calculated. The median value was 2 (0–5) for our combined cohort. To demonstrate the predictive accuracy of the ARS in our patients, we generated a receiver operating characteristic (ROC) curve and calculated the area under the curve (AUC) which was 0.74 (95% CI 0.60–0.88) (Fig. 3).

## Discussion

Previous reports support the efficacy of adrenalectomy for PA in appropriately selected patients. The ARS may be used to predict the clinical success of a patient undergoing adrenalectomy for PA. However, Black and Hispanic populations have a higher rate of HTN and are underrepresented in previous reports. To address these concerns, we present outcomes of adrenalectomy for PA in Black and Hispanic patients. Complete clinical success was obtained in 41.5% of patients, and partial clinical response was achieved in 56.6% of patients. Moreover, 90% of the patients had improvement in their serum potassium exclusive of impact on blood pressure or number of antihypertensives. The prognostic performance of the ARS in the Hispanic and Black population in our cohort was 0.74. This AUC suggests the ARS is an accurate predictor of clinical success following adrenalectomy for PA in the Black and Hispanic population [15].

The increased morbidity and mortality that the diagnosis of PA confers are well described in the literature [16]. Compared with patients with primary HTN, those with PA have a 2.5 × increased risk of stroke and a 1.77 × increased risk of coronary artery disease [17]. Patients with PA also have higher rates of atrial fibrillation, heart failure, metabolic syndrome and diabetes. On the other hand, patients who undergo adrenalectomy have an improvement in their cardiovascular risk profile [18]. Therefore, once a diagnosis of PA is established and its etiology determined, surgical candidates should undergo adrenalectomy [19]. The primary goal of adrenalectomy in PA is to normalize aldosterone, renin, and potassium levels off mineralocorticoid antagonists and improve blood pressure on fewer medications. There are two aspects of curing PA: clinical cure and biochemical cure as proposed by the PASO group [14]. Complete clinical success is accomplished in 27–62% of patients [4]–[9, 14].

As previously mentioned, treatment of HTN in the Hispanic and Black can be difficult given its higher prevalence and reduced response to medications [10, 11]. The outcomes we present after adrenalectomy for PA are similar to other populations previously published [4]-[9, 13].

The ARS was created using a multivariate logistic regression model of variables associated with complete clinical success. The original study evaluated 100 patients from the University of California at San Francisco (UCSF) to create a model and was validated later in a retrospective cohort of 67 patients at Mayo Clinic, Minnesota [4]. Their AUC was calculated at 0.91; however, there is no information regarding the race of the population.

There are preoperative differences as well as variances in the prognostic performance of the ARS between regions, which supports the notion that geographic and genetic factors play a role in the clinical success of adrenalectomy for PA. Utsumi et al. were the first to validate the ARS in the Japanese population [5]. They postulated that given the differences between the American and Japanese populations; it was necessary to validate this model. Using 91 patients, the ARS was found to have an accuracy of 0.81. Aronova et al. assessed long-term accuracy using institutional data from a United States metropolitan population [6]. This population was 59% white and found an AUC of 0.84 with a median follow-up of 3 years. In a larger multicenter study of 7 French University Hospitals, Pasquier et al. validated the ARS [7]. They included 257 patients and the accuracy by AUC was only 0.715. They concluded that the ARS was not predictive of resolution in the French population given genetic and metabolic differences. The clinical success of adrenalectomy for PA was evaluated in the population of Singapore [8]. Loh et al. studied 40 consecutive patients surgically treated for PA in a 10-year period. Similar to our results, the ARS was associated with resolution of hypertension. Finally, the International CONNsortium Study Group conducted the biggest study on the field including 435 patients from 16 medical centers across USA, Europe, Canada and Australia [9]. The overall accuracy was found to be 0.751.

The PASO cohort and the International CONNsortium cohort are currently the biggest and most accurate study cohorts regarding outcomes after surgery for PA and some differences within our study are worth to be mentioned [9, 14]. The BMI in our study is slightly lower to that from the International CONNsortium (27 kg/m2 vs. 29.7 kg/m2) and so is the mean duration of HTN (2.3 vs. 9 years). Our complete success appears to be higher compared to the overall International CONNsortium (27%). However, the outcomes of the Canada and Australia (40% and 38%) subsets are similar to our outcomes. The higher success rate in our cohort and the subsets from the International CONNsortium may be partially explained by lower BMI and/or lower duration of the hypertension.

Burrello et al. [20] created in 2019 the primary aldosteronism surgical outcome score to predict clinical outcomes in PA after adrenalectomy [14]. The PASO score is an alternative to ARS created to predict clinical outcomes. Complete clinical success in our cohort was 41.5%, similar to this report. Therefore, while the PASO score evaluates for important factors including BMI, target organ damage, and largest size nodule, the ARS performs similarly in the Hispanic and Black cohorts.

Interestingly the Black population had a lower percentage of complete clinical success compared to the Hispanic population; however, this difference was not statistically significant. This non-significant difference could be due to the fewer number of Black patients in the cohort. It is of remark that all of the Black patients did undergo AVS during their workup and still the percentage of complete clinical success was lower. Also, of note, the median ARS in the Hispanic population was higher which could potentially explain why this group achieved better complete clinical success. This difference should be further explored in larger studies.

Our study focuses on the Black and Hispanic populations from two referral institutions from different geographic areas. A limitation of this study is selection bias inherit to retrospectives studies. Also, the use of AVS in the workup of PA was not uniform between institutions which may impact outcomes. While we are able to evaluate clinical success and the accuracy of the ARS, our study cannot fully evaluate the biochemical success of each population due to inconsistent reporting of postoperative laboratory values.

Given our small sample size further prospective studies with larger samples are needed to confirm the factors implicated in the lack of complete response in certain groups. In our population, adrenalectomy for PA has efficacy similar to previously published cohorts. ARS remains an accurate model to predict complete clinical success following adrenalectomy in the Black and Hispanic populations and may be utilized preoperatively to inform postoperative clinical outcomes.
